# Male breast cancer: is the scenario changing

**DOI:** 10.1186/1477-7819-6-58

**Published:** 2008-06-16

**Authors:** Kaiyumars B Contractor, Kanchan Kaur, Gabriel S Rodrigues, Dhananjay M Kulkarni, Hemant Singhal

**Affiliations:** 1 Department of Surgery, Oncology, Reproductive Medicine and Anaesthetics, Imperial College, London, UK; 2 Department of Surgery, Northwick Park Hospital, London, UK; 3 Academic Department of Breast Surgery, Nottingham University Hospitals, City Hospital Campus, Nottingham, UK; 4 Department of Surgery, Queen Mary's Hospital, Sidcup, Kent, UK

## Abstract

**Background:**

The overall incidence of male breast cancer is around 1% of all breast cancers and is on the rise. In this review we aim to present various aspects of male breast cancer with particular emphasis on incidence, risk factors, patho-physiology, treatment, prognostic factors, and outcome.

**Methods:**

Information on all aspects of male breast cancer was gathered from available relevant literature on male breast cancer from the MEDLINE database over the past 32 years from 1975 to 2007. Various reported studies were scrutinized for emerging evidence. Incidence data were also obtained from the IARC, Cancer Mondial database.

**Conclusion:**

There is a scenario of rising incidence, particularly in urban US, Canada and UK. Even though more data on risk factors is emerging about this disease, more multi-institutional efforts to pool data with large randomized trials to show treatment and survival benefits are needed to support the existing vast emerging knowledge about the disease.

## Background

Male breast cancer (MBC) comprises about 1% of all breast cancers but is found to be on the rise with the increasing incidence of female breast cancer [1]. The rarity of this condition precludes large randomized trials. Most of the information is therefore based on small single institution retrospective studies or by extrapolation from breast cancer trials in females. In this review we have tried to describe all the available information on male breast cancer with particular emphasis on incidence, etiology, patho-physiology, clinical features, treatment, prognosis and survival to find out if any changing trends are emerging about the disease.

## Methods

An online search was made in Pubmed and MEDLINE databases to find all published studies of interest on male breast cancer. Searches were performed using the terms "breast cancer" and "male". The online cancer incidence database – Cancer Mondial (International Agency for Research on Cancer, Lyon, France) was also searched to provide incidence trends of male breast cancer from 1960–2000. Literature was meticulously reviewed and collated to obtain evidence about various aspects of the disease reviewed under the following sections.

### Epidemiology

**Figure 1:**
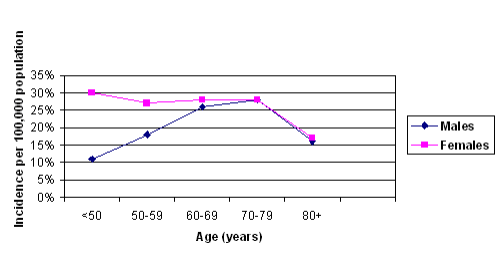
Age wise adjusted incidence of male and female breast cancer (SEER's 12 Registry database, 1992–2000).

Though MBC is rare, a geographic variation in its incidence has been reported. It is higher in USA and UK than in Finland and Japan [2]. National Cancer Institute data on cancer survival in the US shows increase in the incidence of MBC from 0.86 to 1.08 per 100,000 men [1]. An alarming increasing incidence has been seen in the US and Canada whereas it is increasing gradually in other parts of the world as well [3] (Table 1). In the US the highest areas were New York State and California where the incidence has been rising since the 1960s. From data on incidence trends, it seems to be an urban disease in men with high prevalence in these areas. Data from Africa is scanty. In Tanzania, it accounts for 6% while in Zambia it is 15% of all cases of breast cancer [4,5]. Uganda has seen a rising trend in incidence. In Europe, Scandinavian countries like Sweden, Denmark and Finland have been seeing increasing incidences as well. About 240 men are diagnosed in UK each year compared to 40,400 women. There is a documented increase in the incidence of female breast cancer [6,7] over the years as well, the rate of rise faster than male breast cancer. Some studies have even indicated a stable incidence of MBC [8-11]. The prevalence of MBC increases with age. Age frequency distribution for males is unimodal with peak incidence in the late sixth and early seventh decade. By comparison, females have bimodal age frequency distribution with early onset incidence at 50 and late at 70 years. The average age of diagnosis in males is 60 years, which is ten years older than that noticed in female patients with the disease [8,12]. A large database review showed differing trends in age based incidences between male and female breast cancers [13] (Figure 1).

**Table 1:** Changing incidence of male breast cancer. Figures given are Age Standardised Incidence (ASR) per 100,000 population.

**Continent**	**Country**	**Volume 1 1960**	**Volume 4 1975**	**Volume 6 1985**	**Volume 8 1995**	**Volume 9 2000**
North America	USA (New York State)	0.5	1.1	0.8	1.0	1.1
	USA (California)	Na	0.6	0.5	0.6	0.7
	Canada (Alberta)	0.4	0.7	0.6	0.6	0.5
	Canada (Ontario)	Na	Na	0.6	0.6	0.7
						
South/Central America	Columbia	0.3	0.5	0.4	0.1	0.3
	Cuba	Na	0.1	0.2	0.3	Na
	Brazil (Goiania)	Na	Na	0.4	0.7	0.8
						
Europe	UK (South Thames)	0.5	0.4	0.5	0.5	0.6
	Denmark	0.4	0.4	0.5	0.5	0.6
	Sweden	0.4	0.5	0.4	0.4	0.4
	Norway	0.3	0.3	0.5	0.4	0.4
						
Asia	Israel (Jews)	1.4	1.1	0.8	1.4	1.1
	Japan (Osaka)	Na	0.1	0.2	0.2	0.2
	India (Mumbai)	Na	0.3	0.4	0.3	0.4
	China (Shanghai)	Na	0.5	0.3	0.3	0.4
						
Africa	Uganda	0.3	Na	Na	0.1	1.4
	Algeria	Na	Na	0.8	0.7	0.6
	Mali	Na	Na	0.8	0.8	Na
						
Australia	New South Wales	Na	0.7	0.7	0.6	0.7

Note: Na-data not available.

### Etiology and risk factors

The definite etiology of MBC is unknown. Factors such as alteration in hormonal milieu, family history and genetic alterations are known to influence its occurrence. Various studies have shown that conditions that alter the estrogen-testosterone ratio in males predispose to breast cancer [14,15]. Among these conditions the strongest association is with Klinefelter's syndrome. Males with this condition have a fifty times increased risk and accounts for 3% of all MBC [16]. Conditions, which are associated with increased estrogen levels, like cirrhosis [17,18] and exogenous administration of estrogen (either in transsexuals or as therapy for prostate cancer) have been implicated as causative factors [19-22]. Also, androgen deficiency due to testicular disease like mumps, undescended testes, or testicular injury, has been linked to the occurrence of breast cancer in men [23,24]. Occupational exposure to heat and electromagnetic radiation, causing testicular damage and further leading to the development of MBC is also postulated [25,26]. An inherited predisposition for breast cancer is noticed in males-analogous to that in females [27-31]. A positive family history of a first-degree female relative having breast cancer is seen in up to 15–20% patients [32]. This increased risk is conferred by mutations in the breast cancer susceptibility genes (BRCA1 and BRCA2). Mutations in both the BRCA1 and BRCA2 genes are linked to female breast cancer. Genetic studies in males however, have shown that germline mutations in BRCA2 alone account for the majority of hereditary breast cancer [33-36]. No link between BRCA1 and familial breast cancer has been noticed in one study [37], whereas other studies have suggested a possible link [38,39]. The Cambridge study showed that 8% of patients had BRCA2 mutations and all the carriers had a family history of breast, ovarian, prostate or pancreatic cancer [40]. The highest prevalence of BRCA2 mutation in MBC has been noted in Iceland where 40% have the mutation [41]. Several case reports have linked MBC with other genetic disorders like Cowden syndrome [42] and Hereditary Non-Polyposis Colonic Cancer (HNPCC) [43]. It has been recently reported that male breast cancer may also predispose to increased risk of developing a second cancer of the stomach, skin and breast [44].

A strong racial predilection is noted in MBC, with studies establishing a high-risk for Jews. Among them, the Sephardic Jews present at a younger age with advanced stage disease whereas the Ashkenazi Jews have an increased lifetime risk of suffering from the disease [45,46]. Gynecomastia, present in 6–38% of MBC patients has also been implicated as a risk factor [47,48] and some studies have shown positive correlation between the two [49]. An interesting study in the US comparing incidence, pathology and outcomes in male and female breast cancer in a defined population showed more black males than white males to be affected. Also black men with breast cancer had more involved axillary lymph nodes and higher stage than whites at presentation [50]. This is in stark contrast to the high incidence of male breast cancer preponderance in whites as shown in another recently reported study in the US which showed higher incidence in white males, although black males were more not likely to see an oncologist for consideration of chemotherapy and had higher mortality associated with the disease (hazard ratio = 3.29; 95% CI, 1.10 to 9.86) [51]. Reports have shown that an association of MBC and gynecomastia could also represent a chance occurrence as 35–40% of healthy men have clinical or histological gynecomastia [52].

Alcohol has been variably linked as a causative factor in the genesis of MBC. A large Swedish study has not shown any such correlation [53], although it has been implicated as a causal agent in other studies [54]. A case control study conducted in Europe has shown that for alcohol intakes of less than 60 grams per day, the relative risk of MBC is comparable to that in females, and it continues to increase at high consumption levels [55]. Other risk factors mentioned in various studies are low socioeconomic status, obesity, pacemakers, tuberculosis and hyperthyroidism [56,57]. A meta analysis of 7 case-control studies revealed that the risk of MBC to be significantly increased in males with the following characteristics; never married, benign breast disease, gynecomastia, Jewish or history of breast cancer in a first-degree relative [58-61].

### Pathology

The entire spectrum of histological variants of breast cancer has been noted in men. Infiltrating ductal carcinoma is the most predominant subtype with an incidence ranging from 64–93%. The second commonest variant is papillary type seen in 2.6–5% [59-61]. Since the lobular system is not well developed in men, lobular carcinoma is uncommon, although, some cases have been reported in literature [62]. Medullary, tubular, small cell and mucinous carcinoma constitute less than 15% of the cases [63]. Rare tumors like inflammatory carcinomas and sarcomas have also been described [64,65]. Metastasis to breast from tumors of prostate and lung is known [66,67]. In some series most of the tumors were noted to be of high-grade [68] whereas other series have shown predominance of grade 1 and grade 2 tumours [69]. In another study 94% of the tumors were noted to be of grade 1 and 2 [70,71].

Several molecular markers have been identified and studied in MBC patients and include ER (estrogen receptor), PR (progesterone receptor), AR (androgen receptor), p53 gene, Her2neu (Human Epidermal Growth factor-2) expression, gelatinases, p27 gene, MIB-1 (Ki67) index, and Bcl-2 (B-cell lymphoma-2) gene. A high ER positivity as compared to female breast cancer has been noticed consistently in studies on MBC. Approximately 64–85% of cancers in men are ER positive and more than 70% are PR positive [72-74]. Such high levels of positivity may be due to low estrogen levels leaving receptors available for binding and is probably responsible for good hormonal control [75]. A recent study has however shown that like females, the ER positive status does increase with age [76]. Androgen receptor status has been variably reported as being from negative to 95% positive, and its correlation with clinico-pathological factors and survival is not well defined [77]. It has been shown to both stimulate and inhibit growth of AR positive breast cancer lines in vitro [78]. One study proposed that decrease of AR action in the breast might predispose to earlier development of MBC [79]. p53 has been reported to be positive in 21–95% of MBC [80-82]. It is a tumor suppressor gene that regulates cell growth by inducing blockage in the cell cycle. Its over expression has been correlated with recurrence and poorer prognosis in some patients [83] whereas no such correlation has been found in others [84,85]. Levels of ER, PR and AR among MBC patients have been summarized in various studies [1,77,80-82,86,87] (Table 2).

**Table 2:** ER, PR and AR expressions in various studies

**Study**	**Number of patients**	**ER (%)**	**PR (%)**	**AR (%)**
Giordano S *et al.*, [1]	1113	55.3	48.2	Na
Pich A *et al.*, [77]	47	Na	Na	34
Andre S *et al.*, [80]	90	72	74	0
Mourao NettoM *et al.*, [81]	48	75	69	Na
Shpitz B *et al.*, [82]	26	81	Na	Na
Kwiatkowska D *et al.*, [86]	43	61.5	71.8	38.5
Rayson D *et al.*, [87]	77	91	96	95

Note: Na-data not available

#### Her2-neu/c-erbB-2

This is a proto-oncogene, which codes for a tyrosine-kinase transmembrane receptor. Its expression in women is seen in 20–30% of breast cancers and indicates a poor prognosis. In MBC over-expression of Her2-neu correlates significantly with probability of relapse, increased staging, and higher grades of the carcinoma [88-90].

#### Gelatinases

Increased gelatinolytic activity of these enzymes (MMP-2, MMP-9) in MBC patients has been reported in a study to be related to increased tendency to metastasis and poor prognosis [91].

#### p27 and MIB-1

Tumour expression of proliferation marker (MIB-1) and cell cycle related protein (p27) have shown to be good predictors of lymph node metastasis in MBC [92].

#### Bcl-2

Is a proto-oncogene that inhibits apoptosis and helps promote cell growth. In women with breast cancer, studies have shown it to be associated with a favourable prognosis [93,94] but its role in MBC is yet to be defined.

A recent study has evaluated the role of new protein markers p21Waf1 and p27Kip1 as predictors of the most efficient endocrine response [95].

### Clinical features

The typical clinical presentation of breast cancer in 75%–95% of men is a hard eccentric non-tender mass [96]. The mean diameter is reported as 3–3.5 cm but can range from 0.5–12.5 cm. Skin ulceration may be present in a significant number of patients [97]. Collective reviews have shown predilection for the left side in a ratio of 1.07:1. Nipple involvement manifesting as retraction, nipple discharge, fixation or eczema is seen in 40–50% patients. This early presentation of late stage disease is attributed to the small bulk of breast tissue and the central location of these tumours. Paget's disease has been reported in up o 5% of cases. Less common presentations are breast tenderness, itching or symptoms of distant metastasis [98]. Bilateral MBC has been reported in 1.9% patients [99]. Axillary lymph node involvement is very common and clinically suspicious adenopathy has been seen in 40–55% patients. This is explained on the basis of lack of awareness and delayed diagnosis as compared to females. Biologically however, MBC is less aggressive than that in women [100].

A population-based study has shown lymphadenopathy in 37.7% male and 29.2% female patients. Men are 1.6 times more likely to have axillary involvement as compared to females. This study also showed that 6.9% males presented with distant metastasis unlike 5.6% females [101]. The mean duration of symptoms before diagnosis has been reported to be 14–21 months in older studies and 1–8 months in more recent ones [102,103].

### Investigations

The paucity of breast tissue in males makes it difficult to perform and interpret imaging techniques like ultrasound (US) and mammography as compared to females. US has not been shown to be useful in diagnosing MBC [104]. Mammography has however shown to be useful in diagnosing breast cancer in most studies. It forms less than 1% of mammographic examinations done in breast imaging centres [105]. A study of 100 mammograms from Dallas, Texas concluded that sensitivity of mammography was 92%, specificity 90%, positive predictive value 55% and negative predictive value 99% and accuracy of 90% [106]. Mammography can also help to distinguish between benign and malignant lesions of the male breast. The mammographic characteristics in male breast cancer are more likely to show a mass lesion, rather than micro calcifications [105]. Fine needle aspiration cytology (FNAC) is a reliable investigation modality in MBC and helps to differentiate benign from malignant lesions. In a large study (614 cases of males with breast lesions) conducted by John Hopkins Institute USA showed a sensitivity of 95.3%, specificity of 100% and diagnostic accuracy of 98% for FNAC [107]. Other techniques like TC-99m Sestamibi uptake scan have been tried for the diagnosis of malignant masses in males [108].

### Prognosis

**Figure 2:**
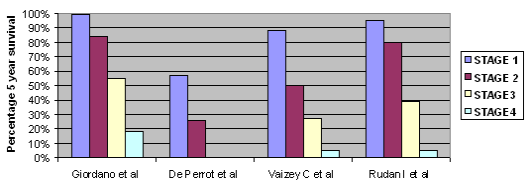
Stage related survival in male breast cancer.

A number of variables have been reported to affect prognosis in MBC patients. Among these, tumour stage [1,109-111] (Figure 2) and axillary nodal status [77,108,112] have consistently been shown to be the most important independent predictors of overall survival. Giordano *et al*., showed [1] five year overall survival rates to be 78% for patients with stage I, 67% with stage II, 40% with stage III, and 19% with stage IV MBC. The five-year survival for patients with node negative disease has been shown in another series to be approximately 70% and that for node positive disease ranging from 37% to 54% [113].

The grade of the tumour has been shown to affect prognosis significantly in univariate analysis. However, the significance of this association is not noted in multivariate analysis [114]. In non-disseminated cases, tumour size and the nodal status were the most important prognostic indicators. Five-year survivals range from 74% for tumours less than 2 cm to 37% for those more than 5 cm in size [115]. One study of 65 MBC cases reported the clinical stage to be the single most significant factor-affecting prognosis irrespective of tumour size or lymph node metastasis [83]. Another study however showed that axillary lymph node status on multivariate analysis was the only prognostic parameter of statistical significance [116]. Though recent studies have provided prognostic information of molecular markers in MBC, the comparative results from these studies are conflicting.

ER and PR positivity is believed to be prognostically favourable in MBC similar to breast cancer in females [78]. However several studies have found that though ER positivity predicted better overall survival in univariate analysis, this difference was no longer significant after adjustments for tumour size, lymph node status and age were made [1,32,83,117]. The role of AR as a prognostic factor is also controversial and most studies have shown a lack of association between AR and survival [77]. In contrast, a recent study has demonstrated that AR expression significantly predicts shorter disease free and overall survival rates [117]. Over expression of c-erbB2 has been associated with shortened survival for patients in some studies [86] whereas others have failed to demonstrate a similar correlation [118-120]. Similarly, p53 mutation has been linked to poor survival and increased local recurrence in some series unlike others where no such link could be shown [83,119]. Similar inconsistent results have also been demonstrated for a variety of other molecular markers like c-myc, MIB-1 DNA ploidy and Her2 neu. BRCA2 associated tumours have been significantly associated with poorer disease free and overall survival rates as was shown in a study where the disease free survival and overall survival for BRCA2 positive patients was 28% and 25% respectively whereas that for controls was 86% and 68% [117].

MBC has traditionally been associated with dismal survival rates as compared to females [27,121]. This is attributed to the late age of presentation and also to delayed detection. In studies where male patients were matched with female patients by stage and age, equivalent overall survival rates have been shown [1,106,122]. Comparison of disease specific survival was shown to have statistically better significant results in males as compared to female breast cancer patients [123]. The disease free, overall and relapse free survival rates in MBC patients is seen to be variable in studies [1,32,60,61,63,109,124-128] (Table 3).

**Table 3:** Survival after treatment of male breast cancer.

**Study**	**Number of patients**	**Period of diagnosis (from-to)**	**5 year overall survival (%)**	**10 year overall survival (%)**	**5 year recurrent free survival (RFS)**
Giordano *et al.*, [1]	2537	1973–1998	63	41	Na
Goss *et al.*, [32]	229	1955–1996	53	Na	47
Cutuli *et al.*, [60]	397	1960–1986	65	38	Na
Donegan *et al.*, [61]	217	1953–1995	50.6	23.7	Na
Borgen *et al.*, [63]	104	1975–1990	88	Na	65
De Perrot *et al.*, [109]	37	1968–1998	Na	44	Na
Herman *et al.*, [124]	65	Na	69.8	59	Na
Hill *et al.*, [125]	142	1973–1994	86	64	Na
Vinod SK *et al.*, [126]	23	1983–1996	66	44	35
Leivonen *et al.*, [127]	42	Na	25	9	Na
Carmalt *et al.*, [128]	42	1958–1996	50	Na	Na

Note: Na-data not available.

### Treatment

There are no prospective randomised trials validating the efficacy of various treatment options in MBC. Management of these patients is based largely on evidence obtained from studies in female breast cancer patients. A literature review shows that there have been marked changes in the treatment protocols for MBC, which mirrors the changes seen in female breast cancer management.

Although radical mastectomy was the treatment of choice in earlier years, less invasive procedures like modified radical mastectomy (MRM) or simple mastectomy are now the standard procedure. A number of series have not shown improvement in survival or local recurrence for male patients who underwent more radical procedures [106,129,130]. Conservative breast surgery in the form of lumpectomy has been reported for Stage-I and ductal carcinoma *in-situ *(DCIS). As in female breast cancer series, lumpectomy alone results in unacceptably high rates of local recurrence, which is reduced upon addition of local radiotherapy (RT) [60]. There is a lack of uniformity in literature regarding the indications and role of postoperative RT in MBC. Interpretation of results from the literature is difficult because most of the studies do not have matched controls for tumour size, nodal status or stage. There have been recommendations for its routine use as it is felt that lack of sufficient breast tissue prevents wide clearance margins on surgery [131,132]. Some studies have even suggested routine inclusion of the internal mammary chain in the radiation field [133]. This approach has however been challenged by others who have shown low local recurrence rates in patients who underwent surgery alone without RT [134,135]. Historically no survival advantage has been noted with the use of adjuvant radiotherapy. Its value is however similar to female situations where a survival benefit has been demonstrated for high-risk patients [136,137].

In an Austrian study, 31 males were irradiated postoperatively to the chest wall and 16 patients to the axilla. Nine patients also received hormone and chemotherapy. 32.2% were Stage II and 35.5% Stage III. Five-year disease free survival was 100% for Stage I, 56.3% for Stage II and 67.3% for Stage III disease. Local relapse occurred in only one patient [126]. Another large German study showed a five-year survival of 59% and 10-year survival of 46% [138]. However no data suggests which patients should receive irradiation to the axilla and which to the chest wall. Therefore a general trend is now noticed towards limiting post mastectomy RT to high-risk patients with advanced T stage and or limited nodal involvement.

The concepts in the management of axillary disease in MBC have changed significantly over the past decade. The standard of care for axillary treatment till now has been axillary lymph node dissection. This is however associated with the attendant risk of significant morbidity. The validation of sentinel lymph node biopsy (SLNB) as an accurate procedure in female patients has prompted similar procedure in men. All of these studies have findings, which compare favourably with the findings of SLNB in females and hence it is being advocated now as the standard surgical procedure in male patients [139,140].

### Adjuvant therapy

#### Hormonal therapy

Due to the high positivity of ER in MBC (75–80%), most cases have an excellent response to hormonal manipulation. Although various methods like orchidectomy, hypophysectomy and adrenalectomy have been described, tamoxifen has shown to have equivalent results as in females. No randomised control trials have been done in male patients and most results have been interpreted using data from female breast cancer patients. No data is available to suggest the duration of treatment in males but one large study showed a 56% disease free survival versus 28% at 5 years in patients of MBC in Stage I and operable Stage 3 disease who were given tamoxifen for 2 years [141]. All these patients were node positive. We feel that more studies need to be done to show whether tamoxifen should be given for 5 years like in women.

#### Systemic chemotherapy

Although no definite data or trials exist about the role and efficacy of adjuvant chemotherapy in MBC, various studies and centre reviews have shown a benefit in survival and prevention of recurrence [142]. A large study involving 24 node positive patients treated with cyclophosphamide, 5-flourouracil and methotrexate showed a five-year actuarial survival of 80% with a median follow up of 46 months [143] and hence it is obvious that chemotherapy is efficacious in node positive men. In a retrospective analysis of therapy in MBC, it was noticed that the median survival of patients who underwent surgery alone was 33 months. However, for those patients who received additional adjuvant therapy in the form of radiation, hormones and chemotherapy, either alone or in combination, the median survival rose to 86 months (p < 0.003). Adjuvant therapy was most effective in large size, node positive and poorly differentiated tumours [129].

### Therapy for metastatic disease

As in females, MBC can spread to the liver, lungs, brain and bones. Rare sites like the choroids plexus and orbit have been documented [144]. There have been cases of metastasis to the breast from a primary in the colon, nasal cavity and from a bronchogenic carcinoma [145].

Hormonal therapy has been proven to help in metastatic disease. Past therapies included orchiectomy, hypophysectomy and adrenalectomy. However these radical and disfiguring procedures have been given up for medications like tamoxifen. A study has mentioned response rates of 32% to 50% for orchiectomy, 17% to estrogens, 43% to steroids, 25% to tamoxifen and 60% to androgens [146,147]. Tamoxifen has shown its beneficial effect in visceral dominant, bone dominant and soft tissue dominant metastasis and the response depends on the degree of ER positivity [148].

Diethylstilbestrol has also been prescribed to patients having soft tissue disease (breast, chest wall and/or lymph nodes) with an overall response rate of 38% [149]. Systemic chemotherapy can be used as a second line of therapy in failed hormonal therapy or in ER negative patients. A study reported response rates of 67% for 5-Flourouracil, doxorubicin and cyclophosphamide, 55% for doxorubicin and vincristine, 53% for cyclophosphamide, 33% for cyclophosphamide, methotrexate and 5-flourouracil, and 13% for 5-flourouracil [150]. Not much has been reported about definitive regimens due to small number of cases.

## Discussion

MBC is a rare disease, which presents mostly in the latter decades of life. It behaves similar to female breast cancer in most ways. There are important risk factors shown like family history and Klinefelter's syndrome. Genetically BRCA-2 mutations are also linked to MBC. 80% of the carcinomas are of the infiltrative ductal variety. Lobular carcinoma is extremely rare although other pathological varieties may be seen. Men have a higher rate of ER positivity, which accounts for good responses with hormonal agents like tamoxifen. They also express markers like Her2-neu, p53 and Cyclin-D1 in similar percentages as females.

Most males present with advanced clinical stage of the disease due to a lack of awareness. Diagnosis is with a mammogram and fine needle aspiration cytology (FNAC) or core biopsy. All patients should be staged completely to exclude metastasis. The treatment of localised disease is by performing a modified radical mastectomy. Adjuvant therapy is mainly tamoxifen as most are strongly ER positive. Chemotherapy may be useful in node positive and locally advanced disease although more evidence is needed for appropriate regimens. The use of adjuvant RT has not been conclusively proven to reduce local recurrence. The treatment of metastatic disease is mainly hormonal which has shown good survival in some studies versus a poor outlook in others. More research and trials have to be conducted to find out the effect of hormonal agents like aromatase inhibitors.

## Conclusion

The scenario in male breast cancer has been changing with respect to its rising incidence-particularly in urban US, Canada and in Uganda. No particular reason for this has been found. Better understanding of the patho-physiology could be possible from emerging data on etiological factors and molecular markers. It is an acceptable fact that the phenotype, pathology, treatment, prognosis and survival of male breast cancer differ in some aspects with that of female breast cancer. Certain impediments like inability to perform randomised trials in male breast cancer due to low incidence of the disease should prompt efforts at setting up large multi-institutional, worldwide studies, so data could be shared and pooled together to enable emergence of meaningful therapies to treat and improve survival. One of the ways forwards may be by setting up a worldwide database of all prevalent cases (a task not difficult in this day and age).

## Competing interests

The authors declare that they have no competing interests.

## Authors' contributions

KC and KK contributed equally to writing the entire manuscript, KC also did database searches, retrieved relevant references and designed various tables and figures.

GR and DK helped in editing the manuscript.

HS helped in the final review and editing of the manuscript.
